# Prognostic Worth of Epidermal Growth Factor Receptor (EGFR) in Patients with Head and Neck Tumors

**DOI:** 10.1155/2020/5615303

**Published:** 2020-11-12

**Authors:** Precious Barnes, F. A. Yeboah, Jinling Zhu, Roland Osei Saahene, Christian Obirikorang, Michael Buenor Adinortey, Benjamin Amoani, Foster Kyei, Patrick Akakpo, Yaw Asante Awuku

**Affiliations:** ^1^Department of Physician Assistant Studies, College of Health and Allied Sciences School of Allied Health Sciences, University of Cape Coast, Ghana; ^2^Department of Molecular Medicine, School of Medical Sciences, College of Health Sciences, Kwame Nkrumah University of Science and Technology, Ghana; ^3^Department of Biology, School of Medical Sciences, Jiamusi University China, China; ^4^Department of Microbiology and Immunology, School of Medical Sciences, College of Health and Allied Sciences, University of Cape Coast, Ghana; ^5^Department of Biochemistry, School of Biological Sciences, College of Agricultural and Natural Sciences, University of Cape Coast, Ghana; ^6^Department of Biomedical Sciences, School of Allied Health Sciences, College of Health and Allied Sciences, University of Cape Coast, Ghana; ^7^Department of Molecular Biology and Biotechnology, School of Biological Sciences, College of Agricultural and Natural Sciences, University of Cape Coast, Ghana; ^8^Department of Pathology, School of Medical Sciences, College of Health and Allied Sciences, University of Cape Coast, Ghana; ^9^Department of Internal Medicine, School of Medical Sciences, University of Health and Allied Sciences, Ghana

## Abstract

**Introduction:**

Head and neck tumors (HNT) are tumors that normally occur at the head and neck region of the body. Epidermal growth factor receptor (EGFR) has been found to be highly expressed in breast and other tumors; therefore, there is the need to investigate the level of EGFR expression among patients with head and neck tumors in Ghana.

**Method:**

The level of EGFR expression was determined in head and neck tumor and control head and neck tissues with quantitative real-time PCR and immunohistochemistry analysis.

**Results:**

The level of EGFR expressions was high in tumor tissues than in the control tissues. There was a significant difference of *p* value 0.025 among the ages >40 and ≤ 40 when the high and low level of EGFR was compared in the head and neck malignant tumor. The area under the curve for the high expression of EGFR among the malignant head and neck tumors was 0.901 with a specificity of 86.4%.

**Conclusion:**

EGFR can serve as a prognostic marker in monitoring patients with HNT as well as a molecular therapeutic target.

## 1. Introduction

Head and neck tumors (HNT) are tumors, which normally occur in the lip, oral cavity, nasal cavity, and nasopharynx, and are considered as the sixth most common tumor globally [1]. The tumor mostly originates from the mucosal surface of the epithelial tissues that line the head and neck regions, and therefore, gene such as EGFR which maintain the integrity of the epithelial tissues needs to be investigated. EGFR is a transmembrane glycoprotein, which is mostly expressed at high levels in many epithelial tumors. It controls important cell functions such as growth, differentiation, motility, and cell death. It consists of extracellular (EC) domain, transmembrane domain (TD), juxtamembrane domain (JD), tyrosine kinase domain (TKD), and carboxyl-terminal tail (CTT). The extracellular domain of the receptor has cysteine-rich regions and is highly glycosylated. This domain has the ability to bind to different ligands such as epigen, amphiregulin, epiregulin, transforming growth factor alpha (TGF-*α*), and betacellulin. The binding of the ligand to the EC domain with another EGFR result in formation of homodimers while binding with other members of the c-ErbB receptor tyrosine kinase family such as epigen, amphiregulin, epiregulin, transforming growth factor alpha (TGF-*α*), and betacellulin results in heterodimers. The EC domain comprises of four subdomains, which are L1, CR1, L2, and CR2. Ligand binding pocket normally occurs because of ligands binding between L1 and L2 subunits. The CR1 and CR2 subdomains contain small modules, which have disulfide bonds and a loop from the back of the CR1 domain. This conformation causes the CR1 domain to bind with other CR1 domain of other receptors, which is important for dimerization. The intrinsic tyrosine kinase activity of the receptor is activated after dimerization. After ligand binding, EGFR is mostly autophosphorylated in different tyrosine residues of the intracellular domain. This provide high affinity sites for different kinds of adaptor molecules, which promote the mitogenic signal to the Ras/MAPK signal transduction pathway. Signalling of Ras causes a multistep phosphorylation cascade, which results in the activation of MAPKs, ERK1, and ERK2 and finally regulates transcription of molecules that initiates cell proliferation, survival, and transformation [2]. EGFR is highly expressed in many cancers such as nonsmall cell lung cancer (NSCLC) [3], breast [4], colorectal [5], prostate [6], gliomas [7], and bladder cancer [8]. The high expression of EGFR has been reported to be related to advanced tumor stages, resistance to therapies, poor prognosis, and increased metastasis [9]. In recent days, specific molecular alterations of a tumor that contribute to the neoplastic phenotype are exploited as targets for therapeutics. Therefore, this study focused on the expression levels of EGFR in head and neck tumors with data evaluated for its significance in disease progression.

## 2. Materials and Methods

A total of 150 paraffin-embedded block samples of HNT (archival tissue specimens) were collected from the pathology laboratory at Cape Coast Teaching Hospital and Komfo Anokye Teaching Hospital (KATH) in Ghana. The tissues were grouped based on the anatomical pattern and further grouped into benign and malignant tumors. The clinicopathological features of the patients are listed in Tables 1 and 2.

### 2.1. Immunohistochemistry

The expression of EGFR was analysed with immunohistochemistry (IHC) technique. Goat anti-EGFR antibody was used. A pathologist subjectively assessed the immunoreaction. The level of EGFR expression was scored semiquantitatively according to the number of positive-staining cells and the staining intensity. Cytoplasmic immunostaining in the tumor tissues was considered positive staining. The results for the expression of EGFR were grouped into 2 categories: low expression (0 to 1+) and high expression (2+ to 3+).

### 2.2. Total RNA Extraction, Real-Time PCR

The total RNA were isolated from the tissues with Trizol reagent (Takara, Otsu, Japan) according to the manufacturer's instructions. Reverse transcription were performed using 0.5 mg total RNA from each tissue. Real-time quantitative polymerase chain reaction (RTqPCR) were performed using the SYBR Green PCR Master Mix (Takara). The sequences of the primer pairs of EGFR were as follows: Forward 5′-GCGTCTCTTGCCGGAATGT-3′and Reverse 5′-GGCTCACCCTCCAGAAGGTT-3′. The experiments were repeated in triplicate. The relative levels of gene expression were represented as ΔCt = CtofEGFR–CtofGAPDH, and the fold change of gene expression was computed using the 2^–*ΔΔ*Ct^ method.

### 2.3. Statistical Analysis

Data analyses were performed using SPSS 17.0 for Windows (SPSS, Chicago, IL). Significance level was set at 5%. For qualitative variables, proportions and percentages were used to summarize the data. The association between EGFR expression and HNT patient clinicopathologic features was evaluated using the *χ*^2^ test.

## 3. Results

**Figure 1:**
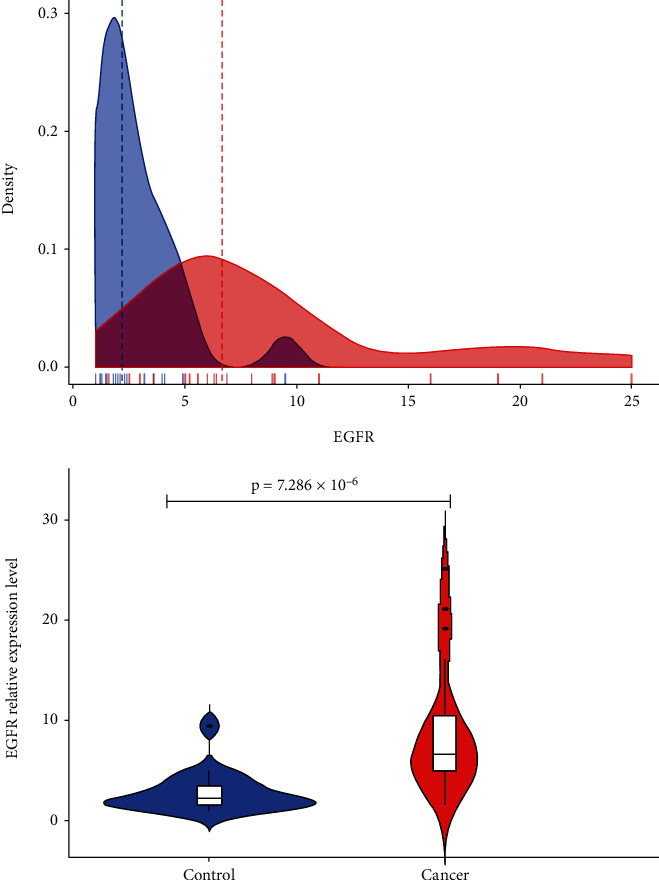
The levels of expression of the EGFR in head and neck tumor tissues were compared with their corresponding head and neck tissues by real-time PCR (polymerase chain reaction).

**Figure 2:**
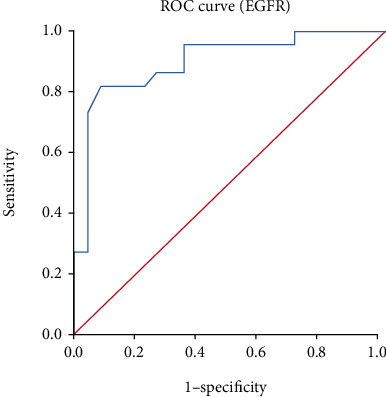
Evaluation of EGFR by ROC curve to determine their sensitivity and specificity.

Figure 1 shows the epidermal growth factor receptor (EGFR) expression levels in the control head and neck tissues compared with the paired head and neck malignant tissues, and Figure 2 depicts the immunohistochemistry expressions of EGFR in tumor tissues. The relative expression of EGFR was significantly higher in tumor compared with control [control: 2.20 (1.45-3.70) vs. case: 6.65 (5.00-11.00); *p*value = 7.286 × 10^−6^] (Figure 1). In comparing clinicopathologic characteristics with EGFR expression both in benign and malignant cases, the relative expression of EGFR was stratified into low and high. A higher proportion of patients with age >40 years (73.5% vs. 26.5%), males (58.8% vs. 41.2%) patients with moderate tumor grade (47.1%), oral cavity tumor (32.4%), and having tumor grade of IV presented with high expression of EGFR. Logistic regression analysis was then used to determine the clinicopathologic characteristics associated with high EGFR expression in head and neck malignant tumors. Age and tumor grade was significantly associated with EGFR expression. Patients with advanced age (>40 years old) [OR = 0.34, 95% CI (0.13-0.85), *p*value = 0.021] presented with significantly lower odds ratio for expressing high EGFR levels, while those with poor tumor grade [OR = 26.56, 95% CI (1.48-476.61); *p*value = 0.026] presented with significantly increased odds ratio for expressing high EGFR levels (Table 2).

A similar risk stratification was performed for patients with benign head and neck tumors. Likewise, patients with advanced age (>40 years old) [OR = 0.23, 95% CI (0.05-0.92), *p*value = 0.038] presented with a significantly lower odds ratio for expressing high EGFR levels, whereas those with poor tumor grade [OR = 10.50, 95% CI (1.02-108.58); *p*value = 0.049] presented with significantly increased odds ratio for expressing high EGFR levels (Table 2).

## 4. Discussion

Overall, there is paucity of data on the levels of expression of epidermal growth factor (EGFR) in head and neck tumors among the African population, particularly in Ghana. This study was therefore designed to bridge the gap in knowledge and to provide relevant data on the expression level of EGFR in head and neck tumors in some selected hospitals in Ghana.

**Figure 3:**
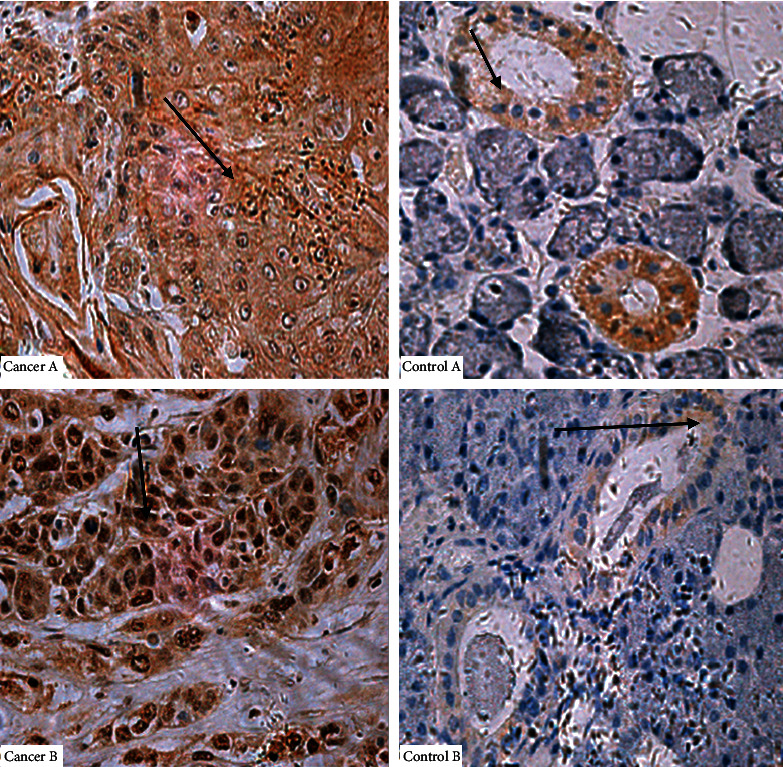
EGFR levels of expression in head and neck control tissues (right) were compared with their corresponding head and neck tumor tissues (left).

**Figure 4:**
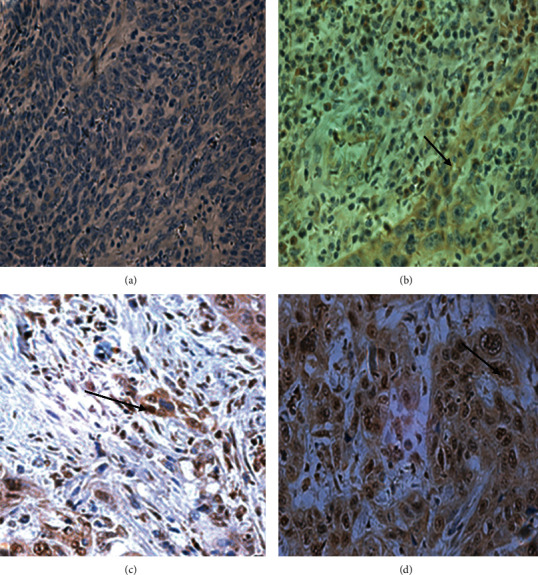
Immunohistochemistry expressions of EGFR in tumor tissues. (a) Negative EGFR (0). (b) Weak levels of EGFR (1+). (c) Moderate levels of EGFR (2+). (d) High levels of EGFR (3+). The data were produced from 3-5 fields per slide. Magnification ×400.

EGFR is a transmembrane tyrosine kinase receptor that is involved in initiating tumor formation when highly expressed [10]. Furthermore, EGFR has the ability to prevent the apoptosis of tumor cells by controlling the basal intracellular glucose level through sodium/glucose cotransporter 1 [11], but this mechanism is normally inhibited when it is highly expressed as a result of cancer. It was observed in the current study that EGFR was highly expressed 68 (69.4) head and neck malignant tissues (Table 2); this is consistent with the study by Wen et al. [12]. They reported 42-80% high expression of EGFR in head and neck tumor tissues. The relative expression of EGFR was significantly higher in tumor compared with control [control: 2.20 (1.45-3.70) vs. case: 6.65 (5.00-11.00); *p*value = 7.286 × 10^−6^] (Figure 3). The EGFR is mostly highly expressed and mutated as a result of toxic environmental stimuli, such as ultraviolet irradiation carcinogens and viral infection. The mutated EGFR can cause the formation of homodimers or heterodimers with other family members, and this can initiate tumorigenesis [13]. Dysregulation of EGFR has also been shown to occur as a result of a high level of expression of EGFR in normal epithelium cells which is close to tumor [14]. This results from an abnormal ligand production and uncontrolled signalling which can also result in the proliferation of tumor cells, angiogenesis, and metastasis. The high levels of EGFR expression in head and neck tumors have also been attributed to gene amplification which further result in a high rate of EGFR transcription and induce high mRNA levels [15]. High levels of EGFR correlates with metastasis in head and neck tumors as a result of the activating mechanism responsible for proteolytic enzyme-matrix metalloproteinases production [16]. From this present study, the EGFR were found in both the nucleus and in the cytoplasm, but it was localized at the cytoplasm of the control tissues (Figures 3 and 4); this is consistent with the findings by Packham et al. [17]. The presence of the EGFR in the cytoplasm shows that EGFR signalling plays a vital role in cell proliferation and is involved in the integrity of the epithelial cells. In normal cells, EGFR is degraded in the lysosomes after activated Ras initiates its signalling pathway. In tumors, however, the EGFR is transported to the nucleus instead of transporting them back to the lysosomes for degradation. The mutated EGFR, which has nuclear localization sequences (NLS), combines with importin *α*/*β*. The complex then joins the nucleoporins, which is found in the nuclear pore complex (NPC), and therefore translocates the EGFR into the nucleus [18]. In the nucleus, EGFR plays a key role in transcription by acting as a transcriptional coactivator for genes, which initiate tumor formation such as cyclin D1. It also activates signal pathways that are involved in the proliferation and differentiation of tumor cells. It also inhibits DNA repair and replication and moreover causes chemotherapy and radiotherapy resistance. From this study, there was a significant difference (*p* value 0.025) between the high and low expression of the EGFR among the ages greater than 40 and less than 40 of the malignant head and neck tissues (Table 2). This was similar to a study by Reimers et al. [19]. From the present study, patients with advanced age (>40 years old) [OR = 0.34, 95% CI (0.13-0.85), *p*value = 0.021] presented with a significantly lower odds ratio for expressing high EGFR levels, while those with poor tumor grade [OR = 26.56, 95% CI (1.48-476.61); *p*value = 0.026] presented with significantly increased odds ratio for expressing high EGFR levels (Table 2). This indicates that high levels of EGFR expression are probably involved in tumor growth and size as suggested by Erin et al. [20]. From the present study, the receiver operating characteristic curve analysis also presented the sensitivity value of the high EGFR expression to be 86.4%, and the area under the curve was also found to be 0.0901 (Table 3). This is similar to a study by Polanska et al. [21]; they reported specificity to be 76.09% and an area under curve of 0.727 of high expression of EGFR in head and neck tumors.

## 5. Conclusion

The results of the present study show that epidermal growth factor influences growth regulation in human head and neck tumors and correlates with prognosis. Epidermal growth factor receptor can serve as a biomarker in diagnosing and prognosis of head and neck tumors. Hence, EGFR can be used as a therapeutic target for the treatment of head and neck tumors.

## Figures and Tables

**Table 1:** Association between EGFR and clinicopathologic characteristics in patients with head and neck malignant tumors.

Variables	Total (*n* = 98)	High EGFR (*n* = 68)	Low EGFR (*n* = 30)	cOR (95% CI)	*p* value
Age					
≤40	33 (33.6)	15 (50.0)	18 (26.5)	1	
>40	65 (66.3)	15 (50.0)	50 (73.5)	2.78 (1.13-6.80)	**0.025**
Sex					
Male	62 (63.0)	22 (73.3)	40 (58.8)	1	
Female	36 (37.0)	8 (26.7)	28 (41.2)	1.93 (0.75-4.94)	0.173
Grade					
Well	36 (36.7)	16 (53.3)	20 (29.4)	1	
Moderate	46 (46.9)	14 (46.7)	32 (47.1)	1.83 (0.74-4.54)	0.193
Poor	16 (16.3)	0 (0.0)	16 (23.5)	26.56 (1.48-476.61)	**0.026**
Tumor site					
Oral cavity	36 (36.7)	14 (46.7)	22 (32.4)	1	
Nasal cavity	28 (28.6)	8 (26.7)	20 (29.4)	1.59 (0.55-4.58)	0.390
Larynx	7 (7.1)	2 (6.7)	5 (7.4)	1.59 (0.27-9.53)	0.607
Mandible	14 (14.2)	3 (10.0)	11 (16.2)	2.33 (0.55-9.87)	0.249
Nasopharynx	11 (11.2)	3 (10.0)	8 (11.8)	1.70 (0.38-7.50)	0.486
Salivary gland	1 (1.0)	0 (0.0)	1 (1.47)	1.93 (0.07-50.76)	0.693
Eye	1 (1.0)	—	1 (1.47)	NA	NA
Tumor stage					
I	25 (25.5)	11 (36.7)	14 (20.6)	1	
II	22 (22.4)	9 (30.0)	13 (19.1)	1.13 (0.36-3.62)	0.831
III	14 (14.3)	2 (6.7)	12 (17.6)	4.71 (0.87-25.61)	0.073
IV	37 (37.8)	8 (26.7)	29 (42.6)	2.85 (0.94-8.66)	0.065

**Table 2:** Association between EGFR and clinicopathologic characteristics in patients with head and neck benign tumors.

Variables	Total (*n* = 52)	Low EGFR (*n* = 19)	High EGFR (*n* = 33)	cOR (95% CI)	*p* value
Age					
≤40	18 (34.6)	3 (15.7)	15 (45.5)	1	
>40	34 (65.4)	16 (84.2)	18 (54.5)	0.23 (0.05-0.92)	0.038
Sex					
Male	28 (53.8)	10 (52.6)	18 (54.4)	1	
Female	24 (46.1)	9 (47.4)	15 (45.5)	0.93 (0.29-2.87)	0.894
Grade					
Well	13 (25.0)	7 (37.0)	6 (18.2)	1	
Moderate	29 (55.7)	10 (52.6)	19 (57.6)	2.22 (0.58-8.40)	0.242
Poor	10 (19.2)	1 (5.3)	9 (27.3)	10.50 (1.02-108.58)	0.049
Tumor site					
Mandible	18 (34.6)	4 (12.1)	14 (42.4)	1	
Nasal cavity	12 (23.1)	5 (26.3)	7 (21.2)	0.40 (0.08-1.97)	0.261
Nasopharynx	3 (5.8)	1 (5.2)	2 (6.0)	0.57 (0.04-8.05)	0.678
Oral cavity	19 (36.5)	9 (47.4)	10 (30.3)	0.32 (0.07-1.33)	0.116

**Table 3:** Diagnostic performance of gene expressions in predicting head and neck malignant tumors.

Gene	Cut-off	AUC	Sensitivity (%)	Specificity (%)	PPV (%)	NPV (%)	*p* value
*EGFR*	≥3.40	0.901	86.4	82.7	78.0	84.2	**<0.001**

AUC: area under curve; SENS: sensitivity; SPEC: specificity; PPV: positive predictive value; NPV: negative predictive value.

## Data Availability

The dataset used is deposited in the archives of the University of Cape Coast Library and is available from the corresponding author on reasonable request.
